# Evaluation of inadequate, indeterminate, false-negative and false-positive cases in cytological examination for breast cancer according to histological type

**DOI:** 10.1186/1746-1596-7-53

**Published:** 2012-05-18

**Authors:** Rin Yamaguchi, Shin-ichi Tsuchiya, Takashi Koshikawa, Toshiro Yokoyama, Kuniko Mibuchi, Yasuhide Nonaka, Sonoe Ito, Hidejiro Higuchi, Mariko Nagao, Koichi Higaki, Jiro Watanabe, Masayoshi Kage, Hirohisa Yano

**Affiliations:** 1 Department of Pathology, Kurume University Medical Center, Kurume, Japan; 2 Department of Pathology, Kurume University School of Medicine, Kurume, Japan; 3 Working Team for the Accuracy of Breast Cytology, Southern Fukuoka Prefecture, Fukuoka, Japan; 4 Department of Diagnostic Pathology, Nippon Medical School Hospital, Tokyo, Japan; 5 Department of Pathology, Aichi Prefectural University, School of Nursing & Health, Aichi, Japan; 6 Department of Diagnostic Pathology, Kurume University Hospital, Kurume, Japan; 7 Department of Pathology, Kurume University School of Medicine, 67 Asahi-machi, Kurume, Fukuoka, 830-0011, Japan

**Keywords:** Breast cytology, False-negative, False-positive, Indeterminate, Inadequate

## Abstract

**Background:**

We previously investigated the current status of breast cytology cancer screening at seven institutes in our area of southern Fukuoka Prefecture, and found some differences in diagnostic accuracy among the institutions. In the present study, we evaluated the cases involved and noted possible reasons for their original cytological classification as inadequate, indeterminate, false-negative and false-positive according to histological type.

**Methods:**

We evaluated the histological findings in 5693 individuals who underwent cytological examination for breast cancer (including inadequate, indeterminate, false-negative and false-positive cases), to determine the most common histological types and/or features in these settings and the usefulness/limitations of cytological examination for the diagnosis of breast cancer.

**Results:**

Among 1152 cytologically inadequate cases, histology revealed that 75/173 (43.6%) cases were benign, including mastopathy (fibrocystic disease) in 38.6%, fibroadenoma in 24.0% and papilloma in 5.3%. Ninety-five of 173 (54.9%) cases were histologically malignant, with scirrhous growing type, invasive ductal carcinoma (SIDC) being significantly more frequent (49.5%) than papillotubular growing type (Papi-tub) (*P* < 0.0001), solid-tubular growing type (*P* = 0.0001) and ductal carcinoma *in situ* (DCIS) (*P* = 0.0001). Among 458 indeterminate cases, 54/139 (38.8%) were histologically benign (mastopathy, 30.0%; fibroadenoma, 27.8%; papilloma, 26.0%) and 73/139 (52.5%) were malignant, with SIDC being the most frequent malignant tumor (37.0%). Among 52 false-negative cases, SIDC was significantly more frequent (42.3%) than DCIS (*P* = 0.0049) and Papi-tub (*P* = 0.001). There were three false-positive cases, with one each of fibroadenoma, epidermal cyst and papilloma.

**Conclusions:**

The inadequate, indeterminate, false-negative and false-positive cases showed similar histological types, notably SIDC for malignant tumors, and mastopathy, fibroadenoma and papilloma for benign cases. We need to pay particular attention to the collection and assessment of aspirates for these histological types of breast disease. In particular, several inadequate, indeterminate and false-negative cases with samples collected by aspiration were diagnosed as SIDC. These findings should encourage the use of needle biopsy rather than aspiration when this histological type is identified on imaging. Namely, good communication between clinicians and pathological staff, and triple assessment (i.e., clinical, pathological and radiological assessment), are important for accurate diagnosis of aspiration samples.

**Virtual slides:**

The virtual slide(s) for this article can be found here: http://www.diagnosticpathology.diagnomx.eu/vs/7349809170055423

## Background

The choice of sampling technique for breast disease has been discussed and might depend on the inadequate diagnostic rates for that methodology [1-3]. For example, the inadequate and indeterminate rates for fine needle aspiration biopsy (FNAB) and needle biopsy should be <10%, as recommended in the Japanese guidelines [4]. However, some Japanese physicians believe that needle biopsy can better avoid inadequate and/or indeterminate results than can FNAB. Nevertheless, whether this is true or not, some of them perform needle biopsy instead of FNAB. On the other hand, increased breast cancer screening is expected. Therefore, low-cost and simple methods are needed for routine procedures, and cytological examination may fulfill these criteria. As the first-line pathological investigation (i.e., symptomatic lesions, confirming of benign lesions), FNAB could be appropriate for breast cancer screening, with the exception of microcalcifications [1].

In such circumstances, we recently reported the accuracy of cytological diagnosis compared with histological diagnosis of breast disease in institutes in our area of southern Fukuoka Prefecture, Japan [5]. We found that the accuracy of cytological diagnosis at these institutes was equivalent to that recommended for diagnostic accuracy in Japanese [4] and UK [6,7] guidelines. It was also equivalent to data reported in other countries [8,9]. However, overall performance differed among the institutes, and was related to the characteristics of these institutes (e.g., the number of cases, number of staff, and specialism).

Furthermore, the number of false-positive and false-negative cases should be as low as possible. To confirm the validity of cytological examinations for breast cancer, managing the accuracy of the examinations is essential. Our earlier study also focused on the differences in the performance of FNAB among institutions.

Therefore, in the present study, we evaluated the inadequate, indeterminate, false-negative and false-positive cases to determine the most common histological types and/or features in these settings, and to demonstrate the usefulness and/or limitations of cytological examination for the diagnosis of breast cancer from the viewpoint of histological types.

## Patients and methods

We conducted a 1-year survey of seven institutes that dealt with many patients, along with a multiple-year survey of institutes with ≤ 100 cases per year. We analyzed data from 2009 and earlier, as previously described [5].

### Classification of cytology and histological samples

In accordance with the General Rules for Clinical and Pathological Recording of Breast Cancer prepared by the Japanese Breast Cancer Society in 2005, individual samples were initially rated as “inadequate” or “adequate”. Samples rated as the latter were graded on a four-category scale: “normal/benign”, “indeterminate” (difficult to distinguish between “benign” and “malignant”), “malignancy suspected”, and “malignant” [4].

**Figure 1:**
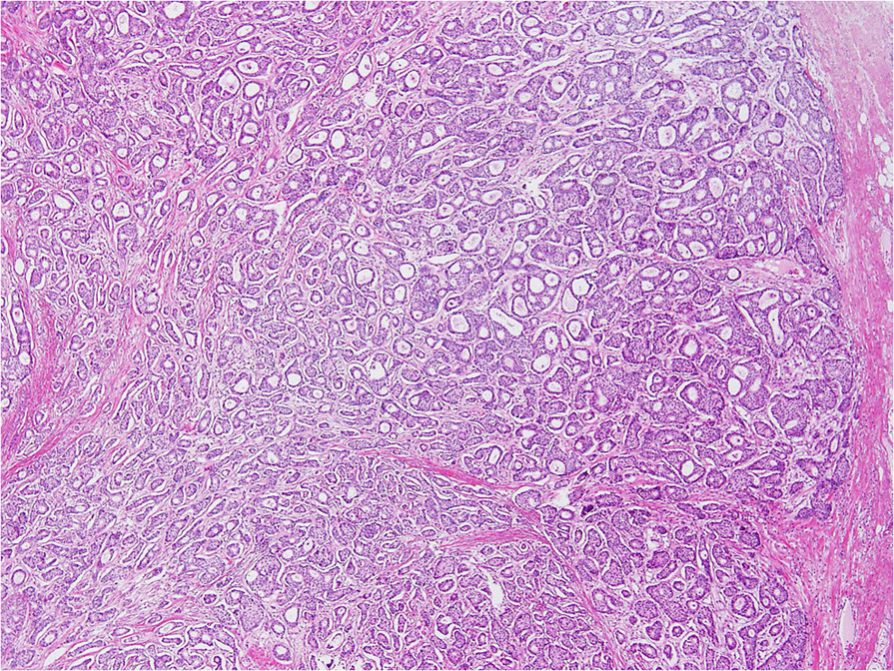
**Representative histopathological specimens of the three subtypes of invasive ductal carcinoma–not otherwise specified.** Papillotubular growing type (Papi-tub) is characterized by the projection of papillae into spaces, and includes cribriform and comedo patterns [4].

**Figure 2:**
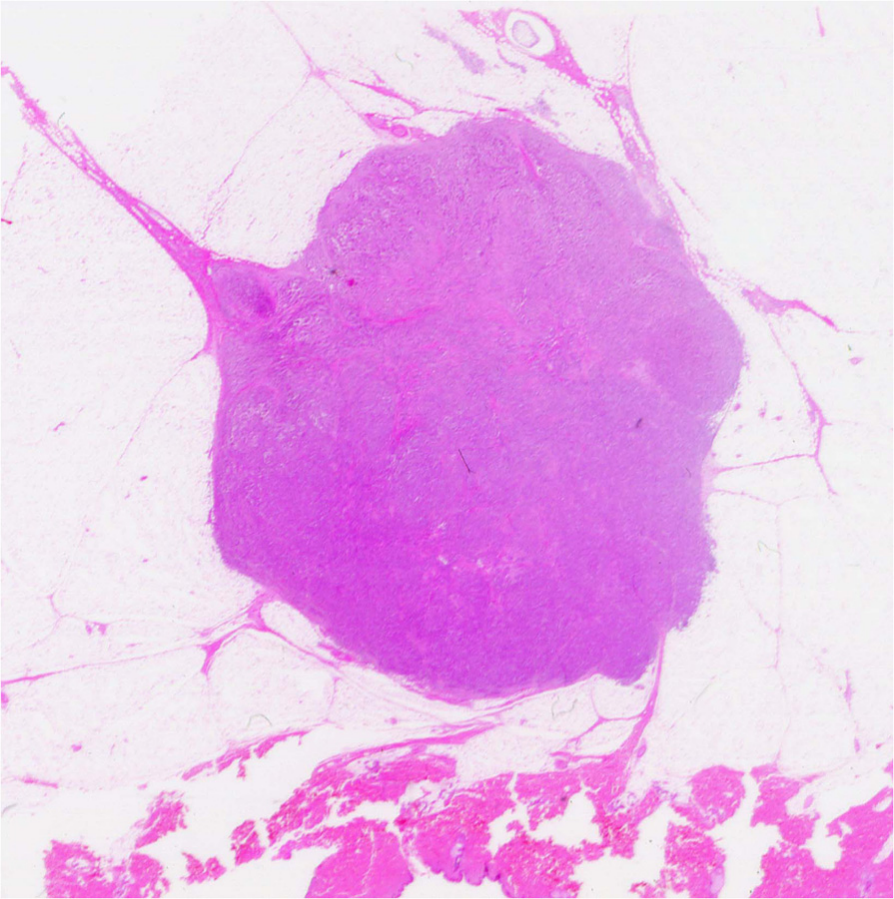
**Representative histopathological specimens of the three subtypes of invasive ductal carcinoma–not otherwise specified.** Solid tubular growing type (Solid-tub) consists of a solid cluster of cancer cells with expansive growths that form relatively sharp borders [4].

**Figure 3:**
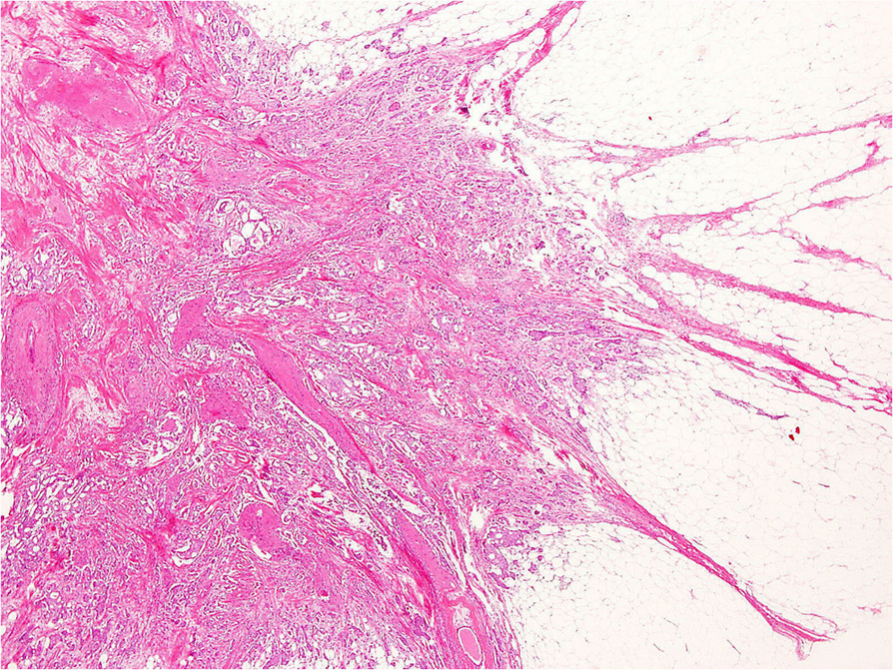
**Representative histopathological specimens of the three subtypes of invasive ductal carcinoma–not otherwise specified.** Invasive ductal carcinoma growing in a scirrhous manner (scirrhous carcinoma; SIDC) is characterized by cancer nests or cells accompanied by marked fibrosis [4].

Invasive ductal carcinoma (IDC) not otherwise specified (NOS) based on the WHO classification [10] was classified into three subgroups based on Japanese Breast Cancer Society guidelines, as follows: (i) papillotubular growing type (Papi-tub), which is characterized by the projection of papillae into spaces, and includes cribriform and comedo patterns [4]; (ii) solid tubular growing type (Solid-tub), which is a solid cluster of cancer cells with expansive growths that form relatively sharp borders [4]; and (iii) IDC growing in a scirrhous manner (scirrhous carcinoma; SIDC), which is characterized by cancer nests or cells accompanied by marked fibrosis [4]. Representative histopathological specimens of these subtypes of IDC-NOS are shown in Figures 1, 2 and 3.

### Variables assessed

Data were collected for 5693 cases. The diagnosis was established histologically in 1250 (22.0%) of these cases, which formed the basis for determining the diagnostic accuracy in our previous report [5]. Inadequate and indeterminate cases based on cytological examinations were confirmed by histological findings, and any cases with discrepancies between the cytological and histological diagnosis (i.e., false-negative and false-positive cases) were analyzed. The assessments reported in our previous study are shown (with permission) in Table 1, including the number of cases in the following categories: inadequate cases, C1 + G1 (benign) and E1 + J1 (malignant); indeterminate cases, C3 + G3 (benign) and E3 + J3 (malignant); false-negative cases, E2 + J2; and false positive cases, C5 + G5. False-negative cases and false-positive cases were reviewed by two investigators (R.Y. and T.Y.) who performed microscopic analyses.

**Table 1:** **Classification of cases and method used for diagnosis (used with permission from [**[5]**])**

		**Histology (B3)**							
**Cytological category**	**(*n*)**	**By operation (B1)**			**By needle biopsy (B2)**				
		**Benign**	**Borderline**	**Malignancy**	**Inadequate**	**Normal or benign**	**Indeterminate**	**Malignancy suspected**	**Malignancy**
Inadequate	A1	C1	D1	E1	F1	G1	H1	I1	J1
	(1152)	(12)	(0)	(82)	(0)	(63)	(3)	(0)	(13)
Normal or benign	A2	C2	D2	E2	F2	G2	H2	I2	J2
	(2914)	(85)	(32)	(40)	(3)	(99)	(2)	(0)	(12)
Indeterminate	A3	C3	D3	E3	F3	G3	H3	I3	J3
	(458)	(27)	(5)	(63)	(3)	(27)	(4)	(0)	(10)
Malignancy suspected	A4	C4	D4	E4	F4	G4	H4	I4	J4
	(272)	(3)	(0)	(65)	(0)	(11)	(0)	(0)	(27)
Malignancy	A5	C5	D5	E5	F5	G5	H5	I5	J5
	(897)	(3)	(0)	(521)	(0)	(0)	(0)	(0)	(35)
Total	A6	C6	D6	E6	F6	G6	H6	I6	J6
	(5693)	(130)	(37)	(771)	(6)	(200)	(9)	(0)	(97)

### Statistical analyses

Differences among histological subtypes in inadequate, indeterminate, and false-negative cases and differences between inadequate and indeterminate cases in terms of histological types were assessed using independent-samples χ^2^ tests for groups with *n* > 5, and Fisher’s exact *p* test for groups with *n* ≤ 5.

## Results

### Analysis of inadequate cases based on cytological diagnosis

**Figure 4:**
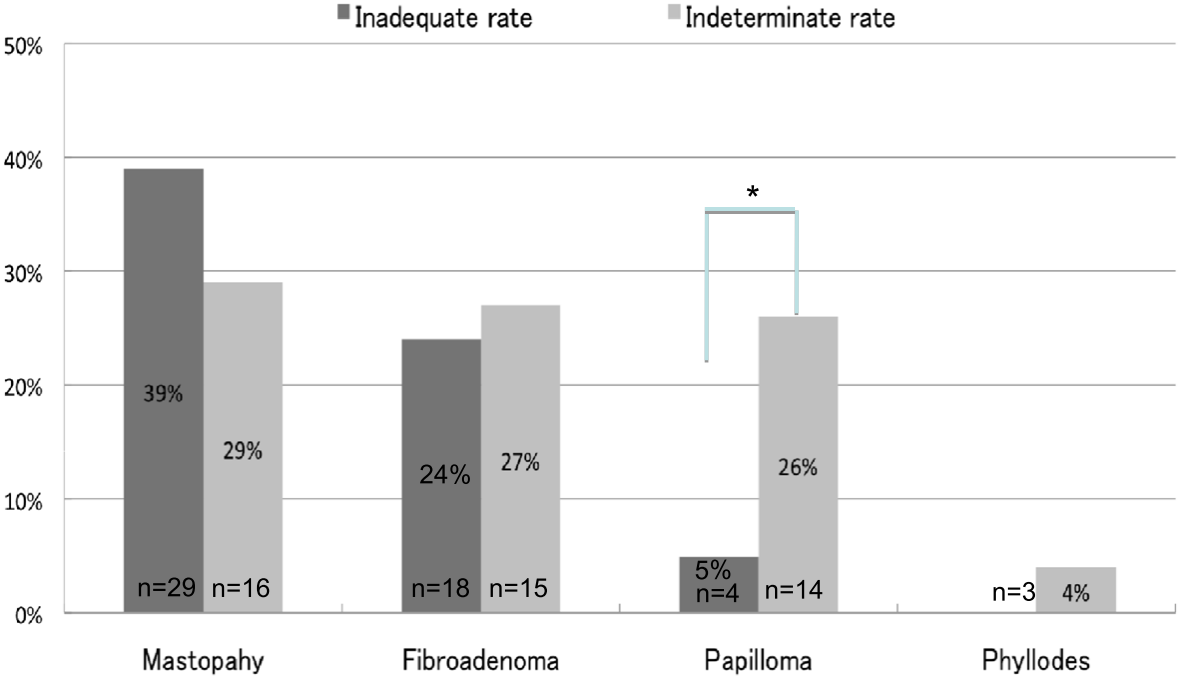
**Final histological diagnosis of benign cases classified as inadequate or indeterminate.** Among inadequate cases, the most frequent histological type was mastopathy (fibrocystic disease) (29/75 cases, 38.6%), followed by fibroadenoma (18/75, 24.0%) and intraductal papilloma (4/75, 5.3%). There were no significant differences in the frequencies of these histological types. Among indeterminate cases, the histological types included mastopathy (16/54, 30.0%), fibroadenoma (15/54, 27.8%) and intraductal papilloma (14/54, 26.0%). Phyllodes tumor was seen in three cases (5.6%). There were no significant differences in the frequencies of these histological types. Papilloma was significantly more frequent in indeterminate cases than in inadequate cases (26% vs 5%; *P = 0.0014). Dark gray, inadequate; light gray; indeterminate. Phyllodes, phyllodes tumor.

Of 5693 cases, 1152 were inadequate (A1) based on cytological examinations. Of these, 173 (15.0%) were confirmed histologically. We first analyzed cases where the sample for cytological examination was classified as inadequate but the final histological diagnosis was benign. Seventy-five of 173 (43.6%) cytologically inadequate cases were classified as C1 + G1 (benign) based on histology. Among these cases, the most frequent histological type was mastopathy (fibrocystic disease) (29/75 cases, 38.6%), followed by fibroadenoma (18/75, 24.0%) and intraductal papilloma (4/75, 5.3%). There were no significant differences in the frequencies of these histological types. The other cases were rated as having benign disease or as benign (24/75, 32.0%) (Figure 4). Overall, 12.0% (9/75) of these cases had a tumor size or hypoechoic area on ultrasound (US) of ≤ 1 cm.

**Figure 5:**
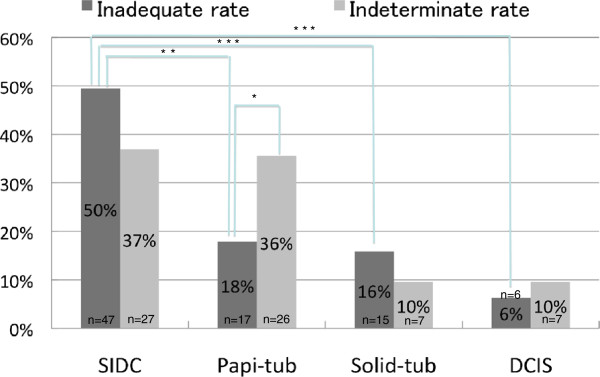
**Final histological diagnosis of malignant cases classified as inadequate or indeterminate.** Among inadequate cases, the most frequent histological subtype was SIDC (47/95, 49.5%), followed by Papi-tub (17/95, 17.9%), Solid-tub (15/95, 15.8%) and DCIS (6/95, 6.3%). SIDC was significantly more frequent than Papi-tub (**P < 0.0001), Solid-tub (***P = 0.0001) and DCIS (***P = 0.0001). Among indeterminate cases, the most common histological types were SIDC (27/73, 37.0%), Papi-tub (26/73, 35.6%), DCIS (7/73, 9.6%) and Solid-tub (7/73, 9.6%). There were no significant differences in the frequencies of these histological types. Papi-tub was significantly more frequent in indeterminate cases than in inadequate cases (36% vs 18%, *P* = 0.0151*). Dark gray, inadequate; light gray; indeterminate. SIDC, scirrhous growing type, invasive ductal carcinoma; Solid-tub, solid-tubular growing type, invasive ductal carcinoma; Papi-tub, papillotubular growing type, invasive ductal carcinoma; DCIS, ductal carcinoma *in situ*.

Next, we assessed cytologically inadequate cases with a final histological diagnosis of malignant. Overall, 95/173 (54.9%) cytologically inadequate cases were classified as E1 + J1 (malignant). Of these cases, > 70% had IDC-NOS. Among these, the most frequent histological subtype was SIDC (47/95, 49.5%), followed by Papi-tub (17/95, 17.9%), Solid-tub (15/95, 15.8%) and ductal carcinoma *in situ* (DCIS) (6/95, 6.3%). The other 10 cases had other malignant histological types, including special types. SIDC was significantly more frequent than Papi-tub (*P* < 0.0001), Solid-tub (*P* = 0.0001) and DCIS (*P* = 0.0001). There were no significant differences in the frequencies of the other subtypes (Figure 5). Overall, 21.1% (20/95) of these cytologically inadequate cases had a tumor size or hypoechoic area on US of ≤ 1 cm.

### Analysis of indeterminate cases based on cytological diagnosis

There were 458 indeterminate cases (A3) based on cytological examinations. Of these, 139 (30.3%) were confirmed histologically. A total of 54/139 (38.8%) histologically indeterminate cases were classified as C3 + G3 (benign). Of these, the histological types included mastopathy (16/54, 30.0%), fibroadenoma (15/54, 27.8%) and intraductal papilloma (14/54, 26.0%). Phyllodes tumor was seen in three cases (5.6%). The other six cases represented other benign histological lesions. There were no significant differences in the frequencies of these histological types (Figure 4). Among these cases, 22.0% (12/54) had a tumor size or hypoechoic area on US of ≤ 1 cm.

Seventy-three of 139 (52.5%) cases were classified as E3 + J3 (malignant). The most common histological types were SIDC (27/73, 37.0%), Papi-tub (26/73, 35.6%), DCIS (7/73, 9.6%) and Solid-tub (7/73, 9.6%). The other six cases were of other malignant histological types. There were no significant differences in the frequencies of these histological types (Figure 5). In 26.0% (19/73) of the cases, the tumor size or hypoechoic area on US was ≤ 1 cm.

### Comparison of histological types between inadequate and indeterminate cases

Regarding benign diseases, papilloma was significantly more common in indeterminate cases than in inadequate cases (26% vs 5%, *P* = 0.0014). By contrast, there were no significant differences in the frequencies of mastopathy or fibroadenoma between inadequate and indeterminate cases (Figure 4). For malignant diseases, Papi-tub was significantly more frequent in the indeterminate cases than in the inadequate cases (36% vs 18%, *P* = 0.0151). The frequencies of SIDC, Solid-tub and DCIS were not significantly different between the inadequate and indeterminate cases (Figure 5).

### Analyses of histological type for false-negative and false-positive cases

**Figure 6:**
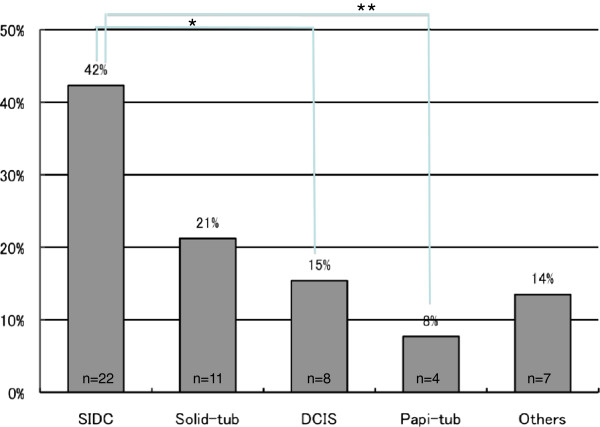
**Final histological diagnosis of false-negative cases based on cytological examination.** SIDC was significantly more frequent than DCIS (*P = 0.0049) and Papi-tub (**P = 0.001). SIDC, scirrhous growing type, invasive ductal carcinoma; Solid-tub, solid-tubular growing type, invasive ductal carcinoma; Papi-tub, papillotubular growing type, invasive ductal carcinoma; DCIS, ductal carcinoma *in situ*.

As shown in Table 1, there were 52 false-negative cases (6.7%), corresponding to E2 + J2. Histologically, these 52 cases consisted of 22 SIDC cases (42.3%), 11 Solid-tub cases (21.2%), eight DCIS cases (15.4%), four Papi-tub cases (7.7%) and seven other cases of special types (13.5%) (i.e., mucinous carcinoma, invasive micropapillary carcinoma). SIDC was significantly more frequent than DCIS (*P* = 0.0049) and Papi-tub (*P* = 0.001). There were no significant differences in the frequencies of the other subtypes (Figure 6).

As shown in Table 1, there were three false-positive cases (0.10%) corresponding to C5 + G5, with the final histological types being fibroadenoma, intraductal papilloma and epidermal cyst. The fibroadenoma was incorrectly rated as positive because there were marked hyperplastic changes of the ductal epithelial cells, which was judged to be malignant atypism. The intraductal papilloma was incorrectly rated as positive because the examiner had limited experience and attached to much importance to the large cell volume. The epidermal cyst was incorrectly rated as positive because atypical squamous epithelial cells with atypical signs (i.e., necrosis accompanied by inflammation) were noted.

## Discussion

In this study, we analyzed cytologically inadequate and indeterminate cases, as well as false-negative and false-positive cases, by evaluating the histological features of these cases.

In cases where the sample was rated as inadequate, SIDC was the most common histological type of malignant tumor, while mastopathy was the most frequent benign disease. It was reported that SIDC cases are often classified as inadequate [11] because SIDC usually consists of abundant fibrous elements, which hinders sample collection [11]. Because tumors ≤ 1 cm in size are relatively infrequent among cases with inadequate samples, it seems likely that the histological type or property (i.e., hard tumor or difficult to collect) is cited more frequently than is tumor size as the reason for rating the sample as inadequate. In 1998, it was reported that tumors of ≤ 1 cm tend to be insufficient [12]. However, we consider that techniques for sample collection have improved since that study.

Among the indeterminate cases with benign diseases, the frequencies of mastopathy, fibroadenoma and papilloma were similar. Among indeterminate cases with malignant diseases, the frequency of Papi-tub was high, nearing that of SIDC. These findings suggest that differentiating between epithelial hyperplastic changes and atypical cancer cells may be difficult. In addition, there are two types of Papi-tub. The first is the papillotubular nodular type, including cribriform carcinoma, and the second mainly consists of intraductal carcinoma components associated with invasive lesions. Both types are generally well differentiated with little cell atypia. The size of tumors in cases rated as indeterminate was not always small, similar to that for inadequate samples. The cases were mainly rated as indeterminate because of tissue features rather than tumor size.

**Figure 7:**
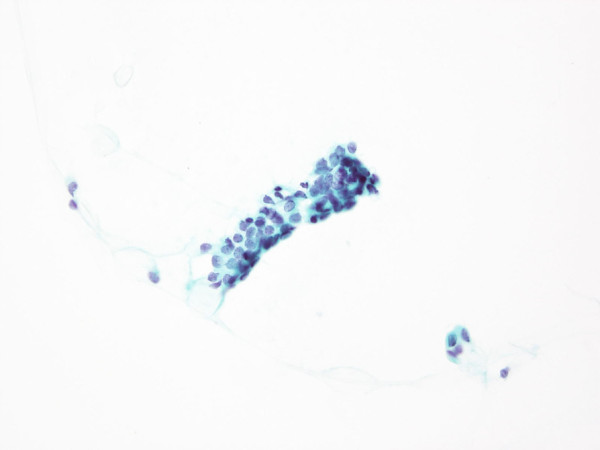
**Representative cytological findings of invasive ductal carcinoma, scirrhous growing type (SIDC).** The cells form small clusters with little atypia on cytological specimens, which hinders attempts to differentiate between benign and malignant cases. This often results in the classification of these cases as indeterminate and/or false-negative. Cases of SIDC are sometimes classified as inadequate when too few cells are collected for evaluation, especially for tumors rich in fibrous components, as these are difficult to sample [12].

The present study revealed that cases with SIDC or mastopathy were likely to be rated as indeterminate or inadequate samples. Cases of SIDC are sometimes classified as inadequate when too few cells are collected for evaluation, especially for tumors rich in fibrous components, as these are difficult to sample [11]. When samples can be extracted, the cells commonly form small clusters with little atypia on cytological specimens, which hinders attempts to distinguish between benign and malignant cases. This often results in the rating of these cases as indeterminate (representative cytological specimens of SIDC as shown in Figure 7). Meanwhile, it may be difficult to obtain samples from cases of mastopathy (fibrocystic disease) and assess hyperplastic changes of the epithelial cells (i.e., ductal hyperplasia). Moreover, some histological types classified as indeterminate differed from those classified as inadequate samples. For example, among benign diseases, papilloma was more frequently classified as indeterminate rather than inadequate. Among malignant diseases, Papi-tub was more frequently classified as indeterminate rather than inadequate.

We found that the most frequent histological type of tumor among the false-negative cases was SIDC. Indeed, cases of SIDC are often reported as false-negatives [11], a finding confirmed by our data. SIDC usually shows with thick fibrosis and, when samples can be extracted, the cells commonly form small clusters with little atypia; this is also why cases with SIDC are often rated as inadequate and indeterminate. Moreover, the atypical cells were relatively small and were similar in appearance to benign or hyperplastic cells.

False-positive cases included cases with papilloma or fibroadenoma, which involve marked hyperplasia of epithelial cells [12,13]. Therefore, particular care is needed during cytology of cases in which a papillary structure or a cribriform pattern is seen. Fibroadenomas are divided into subtypes based on epithelial morphology. For example, the mastopathic type is often mistaken as a malignant tumor based on clinical signs, and the presence of epithelial hyperplasia within the tumor at the time of cytology and needle biopsy [14-16]. In the present study, one fibroadenoma was incorrectly classified as malignant because epithelial hyperplasia within the tumor was incorrectly identified. In this case, however, the fibroadenoma was of a type undergoing marked stromal myxomatous changes (i.e., myxomatous fibroadenoma). We previously reported that the interstitial components of fibroadenoma should receive particular attention during histological analysis [17]. This type of fibroadenoma can be mistaken for a malignant tumor based on US findings, particularly marked growth of the stroma. Therefore, evaluation of the surrounding stroma (e.g., by checking for metachromasia on Giemsa staining) in addition to epithelial cells will help avoid misdiagnosis [17,18].

With the increasing implementation of screening programs in Japan, cytological examination of breast tissue is of increasing importance. Therefore, improving the accuracy of FNAB is an important issue, even though we showed that the accuracy of FNAB is as high as that recommended under the relevant clinical guidelines [5]. We believe that there is room for improvement. For example, it is important to acknowledge that certain histological types, such as SIDC for malignant breast diseases, make tissue aspiration and diagnosis difficult. Indeed, SIDC tended to be diagnosed in cases classified as inadequate, indeterminate and false-negative using aspirated samples in the present study. SIDC was reported to be easy to recognize using appropriate imaging modalities (i.e., mammography and/or US) [11]. Therefore, the present findings should encourage the use of needle biopsy rather than aspiration when this histological type is suspected based on imaging. Furthermore, triple assessment (i.e., clinical, pathological and radiological assessment) should be considered in some cases [5,19]. It is also important that staff receive appropriate pathological and clinical training, and that there is good communication between clinicians and pathological staff [5]. Finally, because there are institutional differences, we recommend that a management system is established to facilitate accurate cytological examination and diagnosis, as we have already reported [5].

## Conclusions

The inadequate, indeterminate, false-negative and false-positive cases showed similar histological types, notably SIDC for malignant tumors, and mastopathy, fibroadenoma and papilloma for benign cases. We need to pay particular attention to the collection and assessment of aspirates. Diagnosis should be reached while considering possible errors in diagnosis for these histological types of breast disease. In particular, several inadequate, indeterminate and false-negative cases with samples collected by aspiration were diagnosed as SIDC. These findings should encourage the use of needle biopsy rather than aspiration when this histological type is identified on imaging.

## Abbreviations

FNAB: Fine needle aspiration biopsy; SIDC: Scirrhous growing type, invasive ductal carcinoma; Papi-tub: Papillotubular growing type, Invasive ductal carcinoma; Solid-tub: Solid tubular growing type, invasive ductal carcinoma; IDC: Invasive ductal carcinoma; NOS: Not otherwise specified; DCIS: Ductal carcinoma *in situ*.

## Competing interests

The authors declare that they have no competing interests.

## Authors’ contributions

RY is the corresponding author and wrote the manuscript. SiT and TK designed the study. TY (cytologist, facility D), KM (cytologist, facility G), YN (cytologist, facility F), SI (cytologist, facility E), EH (cytologist, facility B), MN (cytologist, facility A), JW (pathologist, facility B), KH (pathologist, facility E), and MK (pathologist, facility D) performed the study at each facility. HY provided administrative support and interpreted the data. All authors provided important contributions to the conception and design of the study, reviewed the analytical results, and read and approved the final manuscript.
